# The Role of Platelet-to-Neutrophil Ratio as a Biomarker for Pulmonary Hypertension in Sickle Cell Disease Patients: A Retrospective Cohort Study

**DOI:** 10.3390/medicina62040774

**Published:** 2026-04-16

**Authors:** Abrar J. Alwaheed, Safi G. Alqatari, Sajidah Jaffar Alalwan, Dana Ahmed Alnufaily, Moyad Almuslim, Maryam L. Aldossari, Marj M. Alabdullah, Shahad A. Alzahrani, Abdullah Shaker Al Jama, Hind Asim Kutbi, Rayyan Almusally, Manal A. Hasan, Osama Abdulla Alsultan

**Affiliations:** 1 Department of Internal Medicine, College of Medicine, King Fahd University Hospital, Imam Abdulrahman Bin Faisal University, Dammam 31441, Eastern Province, Saudi Arabia; 2 College of Medicine, Imam Abdulrahman Bin Faisal University, Dammam 31441, Eastern Province, Saudi Arabia; 3 Department of Pharmacology, College of Pharmacy, Imam Abdulrahman Bin Faisal University, Dammam 31441, Eastern Province, Saudi Arabia

**Keywords:** sickle cell disease, pulmonary hypertension, platelet-to-neutrophil ratio, fetal hemoglobin, echocardiography

## Abstract

*Background and Objectives*: Pulmonary hypertension (PH) is a major contributor to morbidity and mortality in sickle cell disease (SCD), yet reliable and accessible biomarkers for cardiopulmonary risk stratification remain limited. This study aimed to evaluate whether the platelet-to-neutrophil ratio (PNR) is independently associated with echo-estimated PH (ePH) in adolescents and adults with SCD and to compare its predictive value with hemoglobin composition and genotype. *Materials and Methods*: A retrospective cohort study was conducted at King Fahd Hospital of the University, Al Khobar, Saudi Arabia (January 2019–January 2025). Clinical, laboratory, and echocardiographic data from 114 patients with confirmed SCD who underwent transthoracic echocardiography (TTE) were analyzed. ePH was defined as tricuspid regurgitant velocity (TRV) ≥ 2.5 m/s or pulmonary artery acceleration time (PAAT) ≤ 105 ms. Multivariable logistic and linear regression models were used to assess associations between PNR, hemoglobin fractions, genotype, and pulmonary pressure estimates. *Results*: Overall, 43% of patients met the criteria for ePH. PNR was not independently associated with ePH or TRV in adjusted analyses. In contrast, higher fetal hemoglobin (HbF) levels were independently associated with lower odds of ePH (adjusted OR 0.92 per 1% increase, 95% CI 0.86–0.98) and lower TRV values. The HbS/β^0^ genotype was significantly associated with increased odds of ePH (adjusted OR 5.44, 95% CI 1.37–24.0). Exploratory analyses demonstrated an inverse association between PNR and lactate dehydrogenase, suggesting that PNR reflects hemolytic activity rather than pulmonary vascular involvement. *Conclusions*: In this retrospective cohort of patients with SCD, PNR was not independently associated with ePH or TRV after multivariable adjustment. In contrast, hemoglobin composition and genotype, particularly higher HbF and the HbS/β^0^ genotype, were significantly associated with pulmonary pressure estimates.

## 1. Introduction

SCD is an inherited hemoglobinopathy caused by a mutation in the β-globin gene that results in the production of abnormal hemoglobin S [1]. The disease is characterized by chronic hemolytic anemia and recurrent microvascular occlusion, leading to progressive multi-organ damage. PH is among the most serious complications of SCD, given its association with impaired cardiopulmonary function, reduced exercise capacity, and increased mortality [1]. Estimates suggest that 10–33% of adults with SCD develop PH based on noninvasive screening modalities [1].

PH in SCD is a heterogeneous and multifactorial disorder and is classified as Group 5 PH in the 6th World Symposium on PH clinical classification [2]. Contributing mechanisms include chronic hemolysis with nitric oxide depletion, endothelial dysfunction, high-output states related to chronic anemia, cardiac remodeling, and thromboembolic phenomena [1,2,3,4]. This complex pathophysiology presents challenges in identifying single biomarkers that reliably reflect pulmonary vascular involvement independent of overall disease severity.

Right-heart catheterization remains the gold standard for the diagnosis and classification of PH; however, TTE is widely used as a noninvasive screening modality to estimate pulmonary pressure probability and guide further evaluation [5,6]. Doppler-derived parameters such as TRV and PAAT are commonly employed to identify patients at increased risk of PH [5,7]. Although practical and accessible, echocardiographic measurements provide probabilistic rather than definitive hemodynamic assessment [5].

Neutrophils and platelets play central roles in the inflammatory and vaso-occlusive processes characteristic of SCD [8]. Neutrophil activation contributes to endothelial dysfunction and microvascular obstruction, while platelets demonstrate persistent hyperactivation that amplifies thromboinflammatory responses [8]. The PNR, a simple and readily obtainable hematologic parameter, has emerged as a potential biomarker reflecting disease severity and risk of complications in SCD and other inflammatory conditions [9,10]. However, its relationship with SCD-associated PH and echocardiographic indices of pulmonary pressure has not been well defined.

Accordingly, this study aimed to evaluate whether the PNR is independently associated with ePH in adolescents and adults with SCD and to compare its predictive value with hemoglobin composition and genotype. To our knowledge, no prior study has examined PNR in relation to echocardiographic markers of PH in SCD while simultaneously accounting for hemoglobin fractions and genotypes. We hypothesized that PNR would demonstrate an independent association with ePH after adjustment for these hematologic factors and sought to determine whether inflammatory indices provide incremental predictive value beyond established determinants of disease severity.

## 2. Materials and Methods

### 2.1. Subjects

This retrospective observational cohort study was conducted at King Fahd Hospital of the University (KFHU), a tertiary care hospital affiliated with Imam Abdulrahman Bin Faisal University (IAU), in Al Khobar, Saudi Arabia, between January 2019 and January 2025.

The study population included adolescents and adults aged ≥14 years with a confirmed diagnosis of SCD based on hemoglobin electrophoresis who underwent TTE during the study period as part of routine clinical care. All eligible patients meeting inclusion criteria during the study period were included in the analysis.

Inclusion criteria comprised the following: (1) confirmed SCD diagnosis, (2) age ≥ 14 years, and (3) availability of echocardiographic parameters relevant to PH assessment, specifically TRV and/or PAAT. Patients were excluded if echocardiographic data were insufficient to estimate pulmonary pressure probability. A total of 114 patients met the inclusion criteria.

Echocardiography in this cohort was not performed as part of a systematic screening program. Rather, TTE examinations were obtained based on clinical indication, including evaluation of dyspnea, suspected PH, history of acute chest syndrome, abnormal cardiopulmonary findings, preoperative assessment, or perceived increased disease severity.

The study was approved by the Institutional Review Board (IRB) of IAU (approval number: IRB-UGS-2024-01-894). This approval covered the retrospective use of patient data collected between January 2019 and January 2025. Given the retrospective nature of the study and the use of anonymized clinical data, the IRB granted a waiver of informed consent. All study procedures were conducted in accordance with applicable ethical guidelines and regulations, and patient confidentiality was strictly maintained throughout the study.

### 2.2. Variables

The primary independent variable was the PNR, calculated by dividing the absolute platelet count by the absolute neutrophil count obtained from the same blood sample.

Additional independent variables included demographic characteristics (age and sex), sickle cell genotype (HbSS and HbS/β^0^), hydroxyurea use, history of blood transfusion, and SCD-related complications (acute chest syndrome, pulmonary embolism, heart failure, and stroke). Laboratory parameters included hemoglobin concentration, white blood cell (WBC) count, platelet count, lactate dehydrogenase (LDH), indirect bilirubin, and hemoglobin fractions (HbS, HbA_2_, and HbF).

The primary dependent variable was ePH, which was defined primarily as TRV ≥ 2.5 m/s, a threshold widely used in SCD cohorts and associated with increased mortality risk [7,11]. In patients in whom TRV was unavailable or not measurable, PAAT ≤ 105 ms was used as an alternative echocardiographic indicator of elevated pulmonary pressures, consistent with contemporary right-heart assessment guidelines [5]. TRV was prioritized when available, and PAAT was used only when TRV could not be reliably measured.

For sensitivity analyses, continuous TRV values were treated as a dependent variable to evaluate linear associations with clinical parameters.

### 2.3. Procedures

Clinical and demographic data were extracted from the hospital electronic medical record system (TrakCare). Laboratory results were obtained from routine clinical records. Efforts were made to exclude laboratory measurements obtained during documented acute vaso-occlusive crises or acute chest syndrome; however, due to the retrospective design, laboratory data were not uniformly collected under strictly defined steady-state conditions. Hemoglobin electrophoresis results were obtained in a non-post-transfusion state to reflect baseline hemoglobin composition.

All echocardiograms were acquired using a GE ultrasound platform (VS60N/70N–VE80/90/95 series) with Application Software version 204 (revision 110.0) and System Software version 204.22.13 (GE HealthCare, Chicago, IL, USA). Examinations were performed by credentialed sonographers and interpreted by board-certified cardiologists. Echocardiographic measurements were extracted from finalized clinical reports. To minimize duplication, each patient was included once in the analysis based on the most clinically relevant echocardiographic assessment within the study period.

### 2.4. Statistical Analysis

Descriptive statistics were reported as numbers and percentages for categorical variables and as medians with interquartile ranges (Q1–Q3) or means ± standard deviations (SDs) for continuous variables, as appropriate. Comparisons according to ePH status were performed using the Wilcoxon rank-sum test for continuous variables and Pearson’s Chi-square test or Fisher’s exact test for categorical variables.

Multivariable logistic regression was used to assess associations between clinical variables and ePH. Age, sex, and PNR were included a priori in adjusted models. Additional covariates were retained based on biological plausibility and univariate association (*p* < 0.20), while limiting the number of predictors relative to outcome events. Adjusted odds ratios (ORs) with 95% confidence intervals (CIs) were reported.

To evaluate the adequacy of the sample size for the primary logistic regression analysis, a post hoc power estimation was performed assuming a two-tailed α = 0.05 and 80% power, with an anticipated PH prevalence of approximately 40%. Under these assumptions, a minimum sample size of approximately 72 participants would be required to detect an odds ratio of about 2.0 for a binary predictor. Although multivariable adjustment and smaller effect sizes may reduce statistical power, the final sample of 114 patients exceeded this estimated threshold, supporting adequate power to detect moderate associations in the primary analyses.

Sensitivity analyses were conducted in the subset of patients with available TRV measurements using linear regression to evaluate associations between TRV and clinical parameters. Exploratory analyses assessed associations between PNR and selected clinical variables using linear regression, excluding platelet and neutrophil counts to avoid collinearity.

Analyses were conducted using R version 4.5.2 (R Core Team 2025, Vienna, Austria) in RStudio version 2025.9.2.418 (Posit Team 2025, Boston, MA, USA). Statistical significance was defined as a two-tailed *p*-value < 0.05.

## 3. Results

### 3.1. Descriptive Statistics

Clinical characteristics of 114 patients were summarized, with statistical comparisons by ePH status. The median age was 31 years; patients with ePH were slightly older than those without ePH. Females comprised 43% of the cohort, and 33% of participants with ePH were female (*p* = 0.08). Laboratory comparisons revealed that patients with ePH had significantly higher platelet counts (mean 470 vs. 361 × 10^9^/L, *p* = 0.012), higher hemoglobin S percentage (83% vs. 78%, *p* = 0.024), and lower hemoglobin F percentage (12% vs. 17%, *p* = 0.006). A history of pulmonary embolism, acute chest syndrome, heart failure, and stroke was more common among those with ePH; however, the number of events did not reach statistical significance. Importantly, echocardiographic measures used to define ePH had consistent results. Patients with ePH had higher TRV (mean 2.77 vs. 2.14 m/s, *p* < 0.001) and lower PAAT (mean 114 vs. 132 ms, *p* = 0.039) (Table 1).

**Figure 1:**
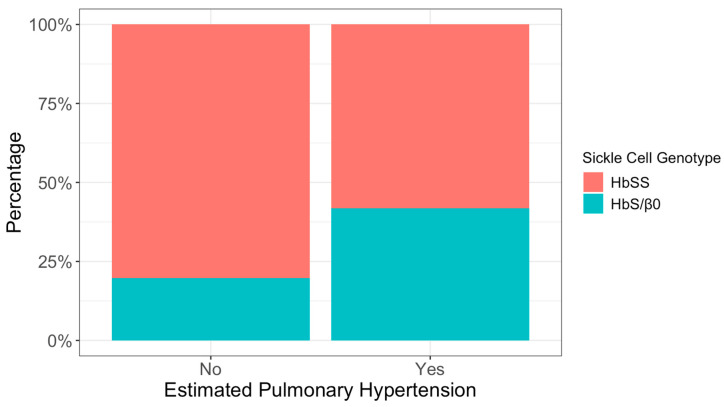
Proportions of echo-estimated pulmonary hypertension by sickle cell genotype.

Homozygous sickle cell genotype HbSS was more common overall (72%) than heterozygous sickle cell beta-zero thalassemia HbS/β^0^ (28%). However, genotype distribution differed significantly by ePH status, with a higher proportion of HbS/β^0^ among patients with ePH (42% vs. 20%; *p* = 0.011) (Figure 1).

### 3.2. Primary Analysis of ePH Associations

In logistic regression analysis, SCD genotype and hemoglobin F fraction were significantly associated with ePH in both the crude and adjusted models. Patients with HbS/β^0^ genotype had higher odds of ePH compared to HbSS (adjusted OR = 5.44, 95% CI: 1.37–24.0, *p* = 0.019). HbF fraction (%) showed an inverse association, with higher levels linked to decreased odds of ePH (adjusted OR = 0.92 per one increase in percentage, 95% CI: 0.86–0.98, *p* = 0.017). Conversely, PNR was not associated with ePH in either crude or adjusted models (Table 2).

**Figure 2:**
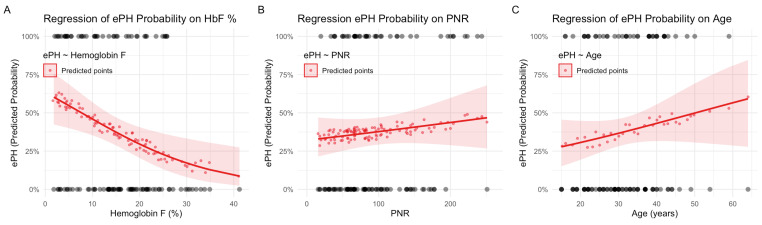
Predicted probability plots of echo-estimated pulmonary hypertension (ePH) status: (**A**) regression of ePH probability on hemoglobin F percentage; (**B**) regression of ePH probability on platelet-to-neutrophil ratio; (**C**) regression of ePH probability on age. Predicited probability of ePH status is represented by the solid red lines; observed binary data points of ePH status are represented by the gray dots at the top and bottom in all plots.

The predicted probability plots illustrate the relationships between key variables and ePH risk. Figure 2A shows that the probability of ePH decreases as HbF % increases, consistent with the inverse association observed in the logistic model. Figure 2B demonstrates a relatively flat relationship between the probability of ePH and PNR values, aligning with the lack of statistical significance for PNR in the logistic models. Figure 2C shows a weak positive linear correlation between ePH probability and age, reflecting the results of logistic analyses (Figure 2).

### 3.3. Sensitivity Analysis of TRV Associations

Since TRV is the primary echocardiographic parameter for screening and assessing pulmonary hypertension risk in SCD patients, a sensitivity analysis was conducted in the subgroup with available TRV measurements to assess its linear correlation with other variables. In adjusted linear regression, HbF % remained strongly and inversely associated with TRV (β = −0.02 per one increase in percentage, 95% CI: −0.03 to −0.01, *p* < 0.001), confirming its protective association. Heart failure history was significantly associated with higher TRV (β = 0.60, 95% CI: 0.11 to 1.1, *p* = 0.017). Age maintained a modest positive association (β = 0.01 per year, *p* = 0.039). Female sex was associated with slightly lower TRV; however, this did not remain statistically significant after adjustment (β = −0.13, *p* = 0.075). PNR was not associated with TRV in either crude or adjusted models (Table 3).

**Figure 3:**
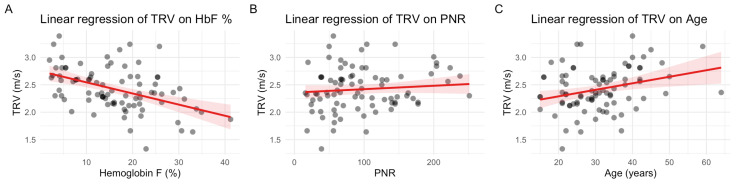
Linear plots of tricuspid regurgitation velocity (TRV): (**A**) linear regression of TRV on hemoglobin F percentage; (**B**) linear regression probability on platelet-to-neutrophil ratio; (**C**) linear regression of TRV on age. Observed data points are represented by the gray dots to show the spread in all plots.

The linear regression plots depict relationships between TRV values and relevant variables. Figure 3A illustrates the strong inverse relationship between HbF % and TRV. Figure 3B shows a flat trend with no apparent correlation between PNR and TRV, consistent with the lack of statistical significance in the linear models. Figure 3C demonstrates a weak positive correlation between age and TRV, suggesting that aging may physiologically increase TRV.

### 3.4. Exploratory Analysis of PNR Associations

The association between PNR and selected clinical parameters was assessed using linear regression. In adjusted models, LDH was inversely associated with PNR (β = −0.08, 95% CI: −0.15 to −0.02, *p* = 0.013), suggesting that increased hemolysis might be linked to lower PNR. HbS % showed a positive association with PNR (β = 1.8, 95% CI: 0.51 to 3.0, *p* = 0.006). WBC count exhibited a borderline negative association (β = −3.5, *p* = 0.090), while hemoglobin concentration showed a non-significant positive trend (β = 6.8, *p* = 0.15). Crude estimates were directionally consistent with adjusted results (Table 4).

## 4. Discussion

### 4.1. Principal Findings

The primary objective of this study was to determine whether the PNR provides independent discriminatory value for identifying ePH in patients with SCD, beyond established hematologic and clinical predictors. In this cohort, PNR was not independently associated with ePH or with continuous TRV measurements after multivariable adjustment. In contrast, hemoglobin composition and genotype emerged as the principal determinants of elevated pulmonary pressure estimates, with higher HbF levels demonstrating a protective association and the HbS/β^0^ genotype associated with significantly increased odds of ePH.

### 4.2. Interpretation of PNR Findings

Although prior studies have suggested that PNR may serve as a marker of disease severity in SCD [9], our findings indicate that this inflammatory index does not appear to reflect pulmonary vascular involvement specifically. Neutrophils and platelets are central contributors to vaso-occlusion, endothelial activation, and thromboinflammatory processes in SCD [8], and reductions in PNR have been associated with acute complications such as stroke and avascular necrosis [9,10]. However, PH in SCD is a multifactorial process involving hemolysis-driven nitric oxide depletion, endothelial dysfunction, vascular remodeling, high-output states, and left-sided cardiac dysfunction [1,2,3]. A simple inflammatory ratio may therefore lack sufficient specificity to capture the complex hemodynamic and structural mechanisms underlying pulmonary vascular disease in this population.

In exploratory analyses, PNR demonstrated an inverse association with LDH, a surrogate marker of hemolysis. This relationship suggests that PNR may more accurately reflect systemic hemolytic or inflammatory burden rather than pulmonary vascular pathology. Chronic hemolysis contributes to nitric oxide scavenging, oxidative stress, and endothelial injury, which are central to the pathophysiology of SCD and its vascular complications [11]. Nevertheless, the absence of association between PNR and echocardiographic markers of pulmonary pressure suggests that its clinical utility for cardiopulmonary risk stratification is limited.

### 4.3. Role of Hemoglobin Composition and Genotype

HbF emerged as a consistent and independent protective factor against elevated pulmonary pressure estimates. Higher HbF levels were associated with lower odds of ePH and lower TRV values in adjusted models. The protective role of HbF in SCD is well established: elevated HbF inhibits hemoglobin S polymerization, reduces erythrocyte sickling, improves rheologic properties, and attenuates hemolysis [12,13]. By limiting intravascular hemolysis, higher HbF levels may mitigate nitric oxide depletion and endothelial dysfunction, processes implicated in pulmonary vascular remodeling and increased pulmonary arterial pressure [11,14]. These findings extend existing knowledge by demonstrating a measurable association between HbF fraction and noninvasive echocardiographic indices of PH risk.

The increased odds of ePH observed among patients with the HbS/β^0^ genotype further reinforce the importance of hemoglobin composition in cardiopulmonary risk stratification. Prior studies have reported a substantial prevalence of PH among patients with HbS/β-thalassemia, with associations to hemolysis and cardiac dysfunction [15]. Severe genotypes may amplify hemolytic stress, myocardial remodeling, and pulmonary vascular injury, thereby increasing susceptibility to elevated pulmonary pressures [15]. Our findings align with these observations and support consideration of genotype as a clinically relevant determinant of cardiopulmonary risk.

### 4.4. Hydroxyurea and Therapeutic Considerations

Hydroxyurea use was not independently associated with ePH in this cohort. The impact of hydroxyurea on PH risk remains incompletely defined. While some studies have demonstrated improvements in hemolysis markers and pulmonary pressure estimates with hydroxyurea therapy [16,17], others have not identified protective effects [15,18]. Variability in treatment adherence, duration of therapy, baseline disease severity, and established vascular remodeling may contribute to these heterogeneous findings. Longitudinal studies incorporating dynamic assessment of HbF response and pulmonary hemodynamics may clarify the extent to which disease-modifying therapy influences pulmonary vascular outcomes.

### 4.5. Prevalence and Clinical Context

The prevalence of ePH in our cohort (43%) is comparable to prior regional echocardiographic studies, including reports from Saudi Arabia demonstrating substantial burden of elevated pulmonary pressure estimates in sickle cell populations [19]. However, echocardiography was performed based on clinical indication rather than systematic screening, which may have enriched the cohort for patients with greater cardiopulmonary burden. The indication-based referral pattern may still limit generalizability to unselected SCD populations. Nonetheless, this design is unlikely to substantially affect the internal associations observed between hemoglobin composition, genotype, and pulmonary pressure indices.

Genetic and clinical characteristics, including severe genotypes and cumulative hemolytic injury, may contribute to this observed prevalence [20]. Although not all clinical complications reached statistical significance, a notable proportion of patients with prior heart failure, stroke, or pulmonary embolism demonstrated elevated pulmonary pressures, underscoring the overlapping and multifactorial nature of cardiopulmonary involvement in SCD.

### 4.6. Limitations and Future Directions

This study has several limitations. First, its retrospective single-center design may limit generalizability and introduces potential for unmeasured confounding. Echocardiography was performed based on clinical indication rather than systematic screening, which may have resulted in selection bias toward patients with greater cardiopulmonary symptoms or disease severity.

Second, PH was defined using echocardiographic parameters rather than right-heart catheterization, the gold standard for hemodynamic diagnosis. Although TRV and PAAT are established screening markers associated with PH risk in SCD [5,7], echocardiography estimates probability rather than confirming definitive pulmonary vascular disease.

Third, laboratory and echocardiographic measurements were not uniformly obtained on the same date, introducing potential temporal variability between hematologic indices and pulmonary pressure estimates.

Finally, although the number of outcome events permitted multivariable modeling, the modest sample size may have limited power to detect smaller effect sizes or subtle associations. Residual confounding cannot be excluded despite statistical adjustment. Larger prospective, multicenter studies with concurrent laboratory and echocardiographic assessments are needed to validate these findings and clarify temporal relationships between hematologic indices and pulmonary pressure estimates. Incorporation of invasive hemodynamic confirmation when feasible would further strengthen future investigations.

## 5. Conclusions

In this retrospective cohort of patients with SCD, the PNR was not independently associated with ePH or TRV after multivariable adjustment. In contrast, hemoglobin composition and genotype, particularly higher HbF and the HbS/β^0^ genotype were significantly associated with pulmonary pressure estimates. These findings suggest that hemoglobin-related factors may provide more clinically informative signals of cardiopulmonary risk than inflammatory cell ratios in this population. Prospective multicenter studies with hemodynamic confirmation are warranted to validate these associations and refine risk stratification strategies.

## Figures and Tables

**Table 1:** Patients’ characteristics with comparison grouped by echo-estimated pulmonary hypertension.

Characteristic	Demographics	Patients Grouped by ePH Status		
	*N* = 114 ^1^	No *n* = 71 ^2^	Yes *n* = 43 ^2^	*p*-Value ^3^
Age (years)	31 (24, 38)	30 (10)	33 (10)	0.14
Sex (female)	49 (43%)	35 (49%)	14 (33%)	0.080
Sickle Cell Disease Type				**0.011**
HbSS	82 (72%)	57 (80%)	25 (58%)	
HbS/β^0^	32 (28%)	14 (20%)	18 (42%)	
Hemoglobin (g/dL)	9.40 (8.60, 10.40)	9.55 (1.60)	9.56 (1.40)	0.7
MCV (fL)	89 (83, 96)	89 (13)	88 (10)	0.2
WBCs (×10^9^/L)	9.4 (7.1, 11.4)	9.1 (3.4)	10.1 (3.7)	0.3
Neutrophils (×10^9^/L)	4.45 (3.00, 5.70)	4.58 (2.31)	5.10 (2.49)	0.3
Platelets (×10^9^/L)	379 (219, 537)	361 (203)	470 (243)	**0.012**
Platelet-to-Neutrophil Ratio (PNR)	81 (57, 129)	94 (78)	107 (63)	0.12
Indirect Bilirubin (mg/dL)	1.17 (0.90, 2.26)	1.73 (1.50)	1.61 (1.23)	0.8
Lactate Dehydrogenase (IU/L)	366 (284, 453)	419 (183)	398 (230)	0.3
Hemoglobin S (%)	81 (75, 87)	78 (13)	83 (7)	**0.024**
Hemoglobin A2 (%)	2.75 (2.10, 3.60)	2.77 (1.23)	3.15 (1.27)	0.050
Hemoglobin F (%)	15 (8, 22)	17 (9)	12 (8)	**0.006**
Pulmonary Embolism (y)	15 (13%)	8 (11%)	7 (16%)	0.4
Acute Chest Syndrome (y)	71 (62%)	43 (61%)	28 (65%)	0.6
Heart Failure (y)	2 (1.8%)	0 (0%)	2 (4.7%)	0.14
Stroke (y)	10 (8.8%)	5 (7.0%)	5 (12%)	0.5
Hydroxyurea (y)	65 (57%)	41 (58%)	24 (56%)	0.8
Transfusion History				0.8
None	3 (2.6%)	2 (2.8%)	1 (2.3%)	
Simple	41 (36%)	28 (39%)	13 (30%)	
Exchange	4 (3.5%)	3 (4.2%)	1 (2.3%)	
Both	66 (58%)	38 (54%)	28 (65%)	
Pulmonary Hypertension (y)	43 (38%)			
Tricuspid Regurgitant Velocity [TRV] (m/s) ^4^	2.36 (2.17, 2.65)	2.14 (0.23)	2.77 (0.23)	**<0.001**
Pulmonary Artery Acceleration Time [PAAT] (ms) ^5^	126 (111, 143)	132 (24)	114 (27)	**0.039**

^1^ Median (Q1, Q3); n (%)—^2^ Mean (SD); n (%); ^3^ Wilcoxon rank-sum test; Pearson’s Chi-square test; Fisher’s exact test; ^4^ *n* = 86 [48 (No), 38 (Yes)]—^5^ *n* = 46 [36 (No), 10 (Yes)]. Abbreviations: ePH = echo-estimated pulmonary hypertension, HbSS = homozygous sickle cell disease, HbS/β^0^ = heterozygous sickle beta-zero thalassemia, MCV = mean corpuscular volume, WBCs = white blood cell count.

**Table 2:** Logistic regression of echo-estimated pulmonary hypertension (ePH) status.

	Crude			Adjusted		
Characteristic	OR	95% CI	*p*-Value	OR	95% CI	*p*-Value
Age (years)	1.03	0.99, 1.07	0.2	1.04	1.00, 1.09	0.076
Sex (female)	0.50	0.22, 1.08	0.082	0.52	0.21, 1.23	0.14
Sickle Cell Disease Type						
HbSS	—	—		—	—	
HbS/β^0^	2.93	1.27, 6.91	**0.012**	5.44	1.37, 24.0	**0.019**
PNR	1.00	1.00, 1.01	0.4	1.00	1.00, 1.01	0.7
Hemoglobin A2 (%)	1.28	0.94, 1.75	0.12	0.57	0.31, 1.00	0.055
Hemoglobin F (%)	0.93	0.89, 0.98	**0.005**	0.92	0.86, 0.98	**0.017**

Abbreviations: CI = confidence interval, OR = odds ratio, HbSS = homozygous sickle cell disease, HbS/β^0^ = heterozygous sickle beta-zero thalassemia, PNR = platelet-to-neutrophil ratio. Age, sex, and PNR are all forced in the adjusted regression model. Other variables were included in the final adjusted model after passing backward selection with the criterion of *p* < 0.20.

**Table 3:** Linear regression of tricuspid regurgitant velocity (TRV).

	Crude			Adjusted		
Characteristic	Beta	95% CI	*p*-Value	Beta	95% CI	*p*-Value
Age (years)	0.01	0.00, 0.02	0.006	0.01	0.00, 0.02	0.039
Sex (female)	−0.21	−0.38, −0.05	**0.011**	−0.13	−0.27, 0.01	0.075
WBCs (×10^9^/L)	0.04	0.01, 0.06	**0.002**	0.02	0.00, 0.04	0.081
PNR	0.00	0.00, 0.00	0.3	0.00	0.00, 0.00	0.8
Lactate Dehydrogenase (IU/L)	0.00	0.00, 0.00	0.4	0.00	0.00, 0.00	0.2
Hemoglobin F (%)	−0.02	−0.03, −0.01	**<0.001**	−0.02	−0.03, −0.01	<0.001
Heart Failure (y)	0.77	0.23, 1.3	**0.006**	0.60	0.11, 1.1	0.017

Abbreviation: CI = confidence interval, WBCs = white blood cell count, PNR = platelet-to-neutrophil ratio. Age, sex, and PNR are all forced in the adjusted regression model. Other variables were only included in the final adjusted regression model after passing stepwise backward selection with a prespecified rule of *p*-value < 0.2.

**Table 4:** Exploratory linear regression of platelet-to-neutrophil ratio (PNR) associations.

	Crude			Adjusted		
Characteristic	Beta	95% CI	*p*-Value	Beta	95% CI	*p*-Value
Hemoglobin (g/dL)	6.1	−2.8, 15	0.2	6.8	−2.5, 16	0.15
WBCs (×10^9^/L)	−2.9	−6.8, 0.87	0.13	−3.5	−7.6, 0.56	0.090
Lactate Dehydrogenase (IU/L)	−0.09	−0.15, −0.02	**0.008**	−0.08	−0.15, −0.02	**0.013**
Hemoglobin S (%)	0.91	−0.27, 2.1	0.13	1.8	0.51, 3.0	0.006

Abbreviation: CI = confidence interval, WBCs = white blood cell count. All variables were tested and only included in the final adjusted regression model after passing stepwise backward selection with a prespecified rule of *p*-value < 0.2.

## Data Availability

The datasets analyzed in this study, along with all resulting analysis reports, can be obtained from the corresponding author upon reasonable request. The data do not contain any identifiable data, and the confidentiality of the included patients is fully maintained.

## Abbreviations

The following abbreviations are used in this manuscript:

**Abbreviation**	**Full Term**
SCD	Sickle Cell Disease
PH	Pulmonary Hypertension
ePh	Echo-Estimated Pulmonary Hypertension
PNR	Platelet-to-Neutrophil Ratio
TRV	Tricuspid Regurgitant Velocity
PAAT	Pulmonary Artery Acceleration Time
TTE	Transthoracic Echocardiography
Hb	Hemoglobin
HbS	Hemoglobin S
HbF	Fetal Hemoglobin
HbA2	Hemoglobin A2
HbSS	Homozygous Sickle Cell Disease
HbS/β0	Sickle Beta-Zero Thalassemia Genotype
IAU	Imam Abdulrahman Bin Faisal University
WBC	White Blood Cell
LDH	Lactate Dehydrogenase
MCV	Mean Corpuscular Volume
OR	Odds Ratio
CI	Confidence Interval
IRB	Institutional Review Board
KFHU	King Fahd Hospital of the University
SD	Standard Deviation
Q1	First Quartile
Q3	Third Quartile
β	Beta Coefficient
